# Monolayer Metasurface Enabling Linear Polarizer and Quarter-Wave Plate for Chip-Scale Atomic Clocks

**DOI:** 10.3390/mi17010025

**Published:** 2025-12-25

**Authors:** Taolong Wang, Zhiqiang Li, Ting Liang, Jiangang Yu, Xiaoqian Cui, Xinpu Li, Zong Yao, Cheng Lei

**Affiliations:** 1 Key Laboratory of Micro/Nano Devices and Systems, Ministry of Education, North University of China, Taiyuan 030051, China; wangtaolongnuc@163.com (T.W.); 20240101@nuc.edu.cn (Z.L.);; 2 Chengdu Spaceon Electronics Co., Ltd., Chengdu 610037, China; 3 North Automatic Control Technology Institute, Taiyuan 030006, China

**Keywords:** metasurface-based LP&QWP, atomic vapor cells, chip-scale atomic clock

## Abstract

A monolayer metasurface-based Linear Polarizer and Quarter-Wave Plate (LP&QWP) is proposed for compact and precise polarization control in chip-scale atomic clocks (CSACs). Finite-difference time-domain simulations reveal that the designed metasurface efficiently converts linearly polarized light into right-handed circularly polarized light. Experimental characterization of devices fabricated on optical glass substrates confirms the polarization manipulation performance, achieving a polarization extinction ratio (PER) of 4.8 dB and a degree of polarization (DOP) of 74.2%, confirming its ability to effectively control the state of polarization. The short-term frequency stability of the developed CSAC prototype reaches 9.29 × 10^−11^ at 1 s and 1.59 × 10^−11^ at 10,000 s, demonstrating its potential for integration into miniature timing systems. The novelty of this work lies in the specific application to CSACs and the co-optimization with attenuation, as the metasurface simultaneously realizes polarization control and optical power balancing within a single functional layer. This study bridges metasurface photonics and atomic frequency standards, providing a functional route toward polarization control and frequency stability in miniaturized chip-scale atomic clocks.

## 1. Introduction

Chip-scale Atomic Clocks (CSACs) based on Coherent Population Trapping (CPT) are compact, low-power, and cost-effective timing devices that can be readily integrated with electronic circuits [1,2,3]. With frequency stability on the order of 1 × 10^−11^ at 10,000 s averaging time, such clocks are attractive for applications in mobile communications, scientific metrology, Positioning, Navigation and Timing (PNT), and seismic monitoring [4,5,6]. A conventional approach to tailoring the State of Polarization (SOP) of light involves combining discrete polarization optics such as Linear Polarizers (LPs) and Quarter-wave Plates (QWPs) [7]. In a typical CSAC configuration, dual-frequency linearly polarized light is converted into circularly polarized states using an LP and QWP (LP&QWP) before interacting with alkali metal atoms [8]. When the frequency difference between the two optical beams matches the hyperfine splitting of the atomic ground states, quantum interference suppresses light absorption, generating a narrow CPT resonance—often referred to as a dark line. This signal is detected by a Photodetector (PD) and used for frequency generation locking [9]. In recent years, small-volume micromachined Atomic Vapor Cells (AVCs) based on alkali metal vapors have attracted considerable attention for enabling further miniaturization of CSACs [10,11,12,13,14,15]. Recent advances in photonic–atomic integrations, such as WGM self-injection-locked lasers [16] and wavelength-stabilized interferometers [17], have demonstrated effective frequency stabilization mechanisms for miniaturized atomic systems. Meanwhile, competing approaches have explored advanced polarization and phase-control strategies, achieving ultrahigh extinction ratios using topological polarization beam splitters [18]; realizing dynamically reconfigured PB optics with ferroelectric nematics [19]; and developing periodically modulated nematic fluids enabling broadband vectorial control [20]. These studies highlight alternative material and structural routes for integration in atomic systems. In this context, our work focuses on CSAC-specific metasurface integration functioning as both LP and QWP, and the co-optimization of polarization conversion and attenuation to enhance CPT performance.

Recent progress in nanophotonics and nanofabrication has accelerated the development of artificial electromagnetic materials with subwavelength architectures, particularly metasurfaces. By engineering the geometry of subwavelength unit cells, metasurfaces provide versatile control over optical wavefronts, including phase, polarization, transmission, and reflection. For instance, adjusting the duty cycle or filling factor of silicon-based subwavelength gratings enables precise tuning of the effective refractive index in waveguides, facilitating accurate manipulation of optical field distributions [21,22]. Such ultrathin, planar optical elements have enabled high-performance integrated devices, including wave plates and polarization converters [23]. Recent advances also include multifunctional polarization metasurfaces [24] and broadband non-diffracting designs for improved beam quality [25]. Moreover, combining metasurface technology with microfabricated alkali vapor cells offers a promising route toward compact photonic packaging, significantly advancing the miniaturization of CSACs [2,26,27].

This study presents the design, simulation, and experimental validation of a multifunctional integrated optical device based on a monolayer metasurface. Through synergistic control of geometric and resonant phases, the proposed structure simultaneously provides linear polarization conversion, quarter-wave plate functionality, and controllable optical attenuation. The device is numerically optimized for operation at the CPT resonance wavelength of alkali atoms. The metasurface is designed with manufacturing compatibility for MEMS atomic vapor cell integration, and its fabrication process demonstrates potential applicability to wafer-level packaging technologies. While full monolithic integration with MEMS vapor cells has not yet been realized, the proposed approach is compatible with low-temperature bonding and atmosphere-controlled sealing processes, which are critical for minimizing alignment errors, thermal stress, and alkali-metal contamination typically encountered in discrete optics (thickness > 3 mm). With a total optical module thickness reduced to the sub-millimeter scale (<0.1 mm), this method offers a compact and scalable pathway toward integrated CSAC physics packages [28,29,30]. The presented design thus provides a feasible and cost-effective optical platform that supports future MEMS-level integration and facilitates the development of miniaturized Positioning, Navigation, and Timing (M-PNT) systems.

## 2. Integrated Chip-Scale Atomic Clock Architecture

**Figure 1:**
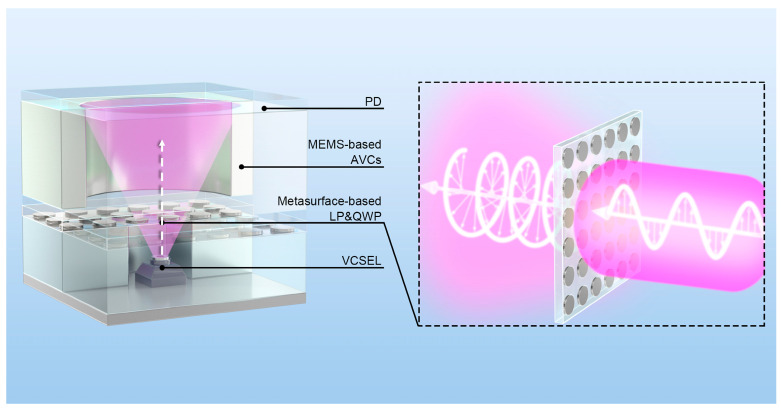
Schematic of the proposed chip-scale atomic clock architecture, which integrates a metasurface-based LP&QWP, a VCSEL, a MEMS-based AVC, and a PD. The metasurface concurrently converts incident linearly polarized light into circularly polarized light and provides optical attenuation.

The architecture of the proposed chip-scale atomic clock is illustrated in Figure 1. It adopts a layered configuration with a Vertical-cavity Surface-emitting Laser (VCSEL) serving as the light source. A key component is the metasurface-based Linear Polarizer and Quarter-wave Plate (LP&QWP), which generates circularly polarized light while simultaneously attenuating the optical intensity [7]. The conditioned beams then propagate through the MEMS-integrated atomic vapor cell, and the resulting CPT resonance signal is detected by a PD. A feedback loop between the PD and VCSEL stabilizes the output, ensuring the generation of a precise time-frequency signal.

## 3. Design of Metasurface-Based LP&QWP

The design of the metasurface-based LP&QWP relies on engineering the complex transmission responses along two orthogonal principal axes of the anisotropic meta-atom by tailoring its geometry and orientation. The polarization transformation is modeled using Jones calculus [31]. The incident field is written as the Jones vector

(1) $$E_{\mathrm{in}}=\left[\begin{array}{l} E_{x}\\ E_{y} \end{array}\right]$$

Let $\left(u,v\right)$ denote the principal axes of the ellipse (long/short axes). In this eigen-axis basis, the metasurface is modeled as a linear anisotropic element without eigen-axis coupling:

(2) $$J_{un}=\left[\begin{array}{cc} t_{u} & 0\\ 0 & t_{v} \end{array}\right],\;t_{u}=\left|t_{u}\right|e^{i\phi u},t_{v}=\left|t_{v}\right|e^{i\phi v}$$

where $t_{u}$ and $t_{v}$ are complex transmission coefficients (including loss). In general, $\left|t_{u}\right|\neq \left|t_{v}\right|$ for lossy metal/dielectric stacks (e.g., Cr/Ag).

If the meta-atom is rotated by an angle α with respect to the laboratory axes $\left(x,y\right)$, the Jones matrix in the lab frame is obtained by a similarity transformation:

(3) $$J_{xy}(\alpha )=R(-\alpha )J_{uv}R(\alpha ),R(\alpha )=\left[\begin{array}{cc} \cos\alpha & -\sin\alpha \\ \sin\alpha & \cos\alpha \end{array}\right]$$

Expanding Equation (3) gives

(4) $$J_{xy}(\alpha )=\left[\begin{array}{cc} t_{u}\;\cos^{2}\alpha +t_{v}\;\sin^{2}\alpha & {(t}_{v}-t_{u})\sin\alpha \cos\alpha \\ {(t}_{v}-t_{u})\sin\alpha \cos\alpha & t_{u}\;\sin^{2}\alpha +t_{v}\;\cos^{2}\alpha \end{array}\right]$$

Equation (4) shows that cross-polarized terms in the $\left(x,y\right)$ basis generally appear after rotation ($J_{xy}=J_{yx}\neq 0$ for $\alpha \neq 0^{\circ },90^{\circ }$) even though the element is diagonal in $\left(\omega ,\upsilon \right)$. Therefore, assuming $t_{xy}=t_{yx}=0$ in the laboratory frame is not generally valid.

For an x-polarized incident wave $E_{\mathrm{in}}={\left[1,0\right]}^{T}$, the transmitted field is

(5) $$E_{\mathrm{out}}=J_{xy}(\alpha )\left[\begin{array}{c} 1\\ 0 \end{array}\right]=\left[\begin{array}{c} t_{u}\;\cos^{2}\alpha +t_{v}\;\sin^{2}\alpha \\ {(t}_{v}-t_{u})\sin\alpha \cos\alpha \end{array}\right]$$

Setting α=π/4 yields

(6) $$E_{\mathrm{out}}(\frac{\pi }{4})=\frac{1}{2}\left[\begin{array}{l} t_{u}+t_{v}\\ t_{v}-t_{u} \end{array}\right]$$

Define the phase retardance between the two eigen-axes as

(7) $$\Delta \phi \equiv \phi_{v}-\phi_{u}$$

Ideal conversion to circular polarization requires equal-amplitude orthogonal components with a $\pm \frac{\pi }{2}$ phase difference. In the present (generally lossy) case, this condition can be written directly from Equation (6) as

(8) $$\left|t_{u}{+t}_{v}\right|=\left|t_{v}-t_{u}\right|,\arg(t_{v}-t_{u})-\arg(t_{u}{+t}_{v})=\pm \frac{\pi }{2}$$

As a useful special case, if $\left|t_{u}\right|\approx \left|t_{v}\right|$ and $\Delta \phi \approx \pm \frac{\pi }{2}$, the metasurface approximates a quarter-wave plate when rotated by $\alpha =45^{\circ }$. When $\left|t_{u}\right|\neq \left|t_{v}\right|$ common in (Cr/Ag), the output is generally elliptically polarized rather than perfectly circular; nevertheless, $\Delta \phi \approx \pm \frac{\pi }{2}$ still maximizes circularity, while the amplitude imbalance $\frac{\left|t_{u}\right|}{\left|t_{v}\right|}$ determines the residual ellipticity.

**Figure 2:**
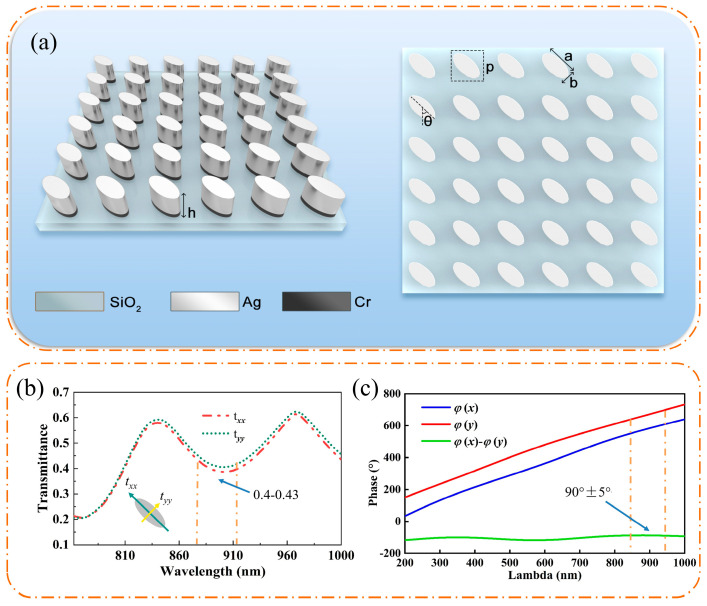
Schematics of the metasurface-based LP&QWP: (**a**) side view and top view. Optical transmission characteristics as functions of wavelength for y-polarized incident light: (**b**) transmittance spectra and (**c**) phase shift.

The structure of the proposed metasurface-based LP&QWP is shown in Figure 2a. It consists of elliptical metal cylinders (Cr/Ag, with a total height of 60 nm: 10 nm Cr and 50 nm Ag) on a transparent silica (SiO_2_) substrate. The cylinders are arranged with a uniform period of 540 nm in both x and y directions to suppress non-zero-order diffraction around the target wavelength of 894 nm.

To determine the optimal geometrical parameters, we employed a coarse-to-fine parameter sweep strategy, in which a manual tuning process guided by FDTD simulation results was performed over the ellipse major and minor axes (a, b) and the lattice period p, with the meta-atom rotation fixed at Φ = 45° for PB-type operation. A coarse grid search was first carried out to locate the region exhibiting near-quarter-wave behavior, followed by a refined parameter scan with smaller step sizes around the optimal region while simultaneously monitoring both the phase and amplitude responses. During optimization, the lattice period p was constrained to remain subwavelength with respect to the operating wavelength (around 894.6 nm) and the refractive index of the surrounding medium, suppress higher diffraction orders and reduce unwanted artifacts that could degrade polarization conversion and phase uniformity in the CSAC optical setup.

Optimized parameters for the metal cylinders include semi-axes of a = 490 nm and b = 205 nm, and a rotation angle of θ = 45°, which collectively yield high transmittance and the desired phase shift. The optical performance was evaluated using finite-difference time-domain (FDTD) simulations performed with Ansys Lumerical. A linearly polarized plane wave, polarized along the y-direction and propagating upward along the *z*-axis from the SiO_2_ substrate, was used as the source over a wavelength range of 800–930 nm. Periodic boundary conditions were applied in the x and y directions, and a perfectly matched layer (PML) was used in the z-direction. The transmittance and phase shift of the output wave after passing through the metasurface were subsequently calculated.

The transmittance spectra and phase of the output waves are plotted as functions of wavelength in Figure 2b and Figure 2c, respectively. The results indicate minimal attenuation of the incident light across the investigated spectrum. Notably, as highlighted by the green line in Figure 2c, the proposed metasurface maintains a consistent phase retardation of π/2 (90°) between the transmitted field components along the two principal axes across the operational band. Crucially, this stable π/2 phase retardation enables nearly uniform polarization conversion efficiency throughout the specified wavelength range.

## 4. Experimental Results

### 4.1. LP&QWP Manufacturing

**Figure 3:**
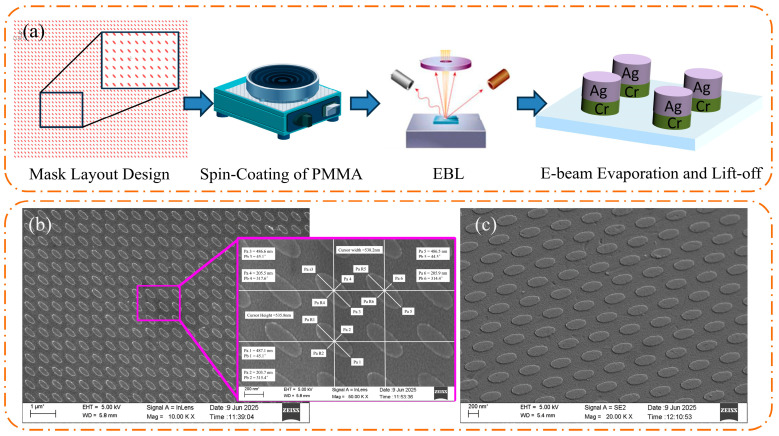
(**a**) Fabrication workflow; (**b**,**c**) SEM images of the fabricated metasurface at different magnifications.

The metasurface structures were fabricated via electron-beam lithography (EBL) and plasma etching. The process began with the design of a .tdb layout file in L-Edit that confirmed to the target specifications. A SiO_2_ substrate was spin-coated with a thin layer of poly (methyl methacrylate) (PMMA) electron-beam resist and baked at 120 °C for 5 min. The metasurface pattern for the quarter-wave plate was then exposed using a JBX-6300FS EBL system. Following exposure, the sample was developed in a MIBK: IPA (1:3) solution and rinsed in ethanol. A bilayer metal stack—10 nm of chromium as an adhesion layer and 50 nm of silver—was then deposited via electron-beam evaporation. The sample was then cleaned with acetone and ethanol to remove residual resist ensuring a clean surface. This procedure resulted in a Cr/Ag metasurface patterned on a glass substrate with the intended LP&QWP functionality. The overall fabrication workflow and a representative scanning electron microscopy (SEM) image of the fabricated metasurface are presented in Figure 3, confirming the successful realization of the target nanostructures.

### 4.2. Metasurface-Based LP&QWP Performance Analysis

**Figure 4:**
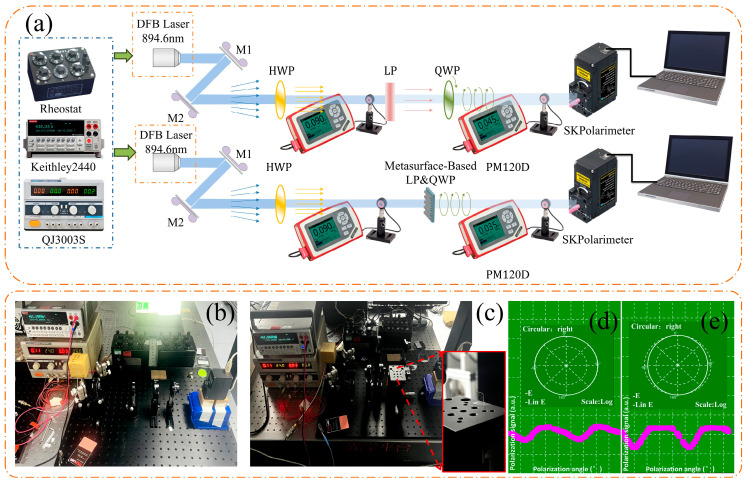
Optical test platform: (**a**) schematic diagram; (**b**) experimental setup for conventional glass-based optics; (**c**) setup for the metasurface-integrated LP&QWP; (**d**) polarization profile and optical power for the conventional LP + QWP combination; (**e**) polarization profile and optical power for the metasurface LP&QWP.

An optical test platform was assembled to evaluate the performance of the metasurface device, all illustrated in Figure 4. The system consisted of a distributed feedback (DFB) laser source operating at 894.6 nm, steering mirrors, a half-wave plate (HWP), a linear polarizer (LP), a conventional quartz quarter-wave plate (QWP), the metasurface-based QWP, a polarization analyzer (SK Polarimeter, Hamburg, Germany), DC power supplies (Keithley 2440, Cleveland, OH, USA and QJ3003S, Ningbo, China), a variable resistor, and an optical power meter (Thorlabs PM120D, Newton, NJ, USA). This setup enabled direct comparison of polarization conversion and optical attenuation between the conventional and metasurface-based LP&QWP configurations. In both cases, linearly polarized light from the DFB laser was converted into circularly polarized light after transmission through the respective LP&QWP assembly.

Polarization measurement results in Figure 4d,e and Table 1 indicate that the metasurface-based LP&QWP achieves right-handed circular polarization with a polarization extinction ratio (PER) of 4.8 dB and a degree of polarization (DOP) of 74.2%, comparable to the conventional LP + QWP configuration (PER = 4.9 dB, DOP = 80.2%). Under identical illumination, the conventional LP + QWP attenuates an incident optical power of 90 μW to 45 μW, whereas the metasurface-based LP&QWP further reduces it to 35 μW, thereby integrating polarization conversion and optical attenuation into a compact architecture. The present experiments primarily validate the metasurface’s equivalent quarter-wave plate (QWP) polarization conversion behavior together with its intrinsic insertion loss. It should be noted that the circular polarization verification is restricted to the 894.6 nm CPT resonance wavelength.

### 4.3. Atomic Clock Performance Analysis

**Figure 5:**
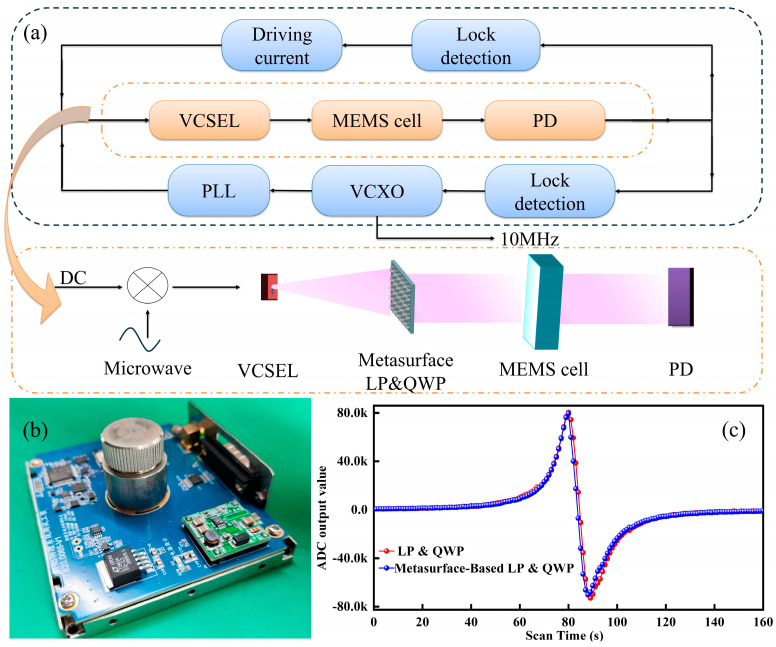
(**a**) Chip atomic clock measurement schematic; (**b**) Proposed chip scale atomic clock; (**c**) CPT microwave demodulation results Comparison.

A dedicated optical setup was used to evaluate the clock prototype, as shown in Figure 5a. It consisted of a DFB laser, the metasurface LP&QWP, a polarization beam splitter, a MEMS-based atomic vapor cell, a PD, and an oscilloscope. The DFB laser emitted at 894.6 nm, and the light was converted to circular polarization by the metasurface before passing through the vapor cell. The transmitted signal was detected by the PD and recorded on the oscilloscope. Temperature stability of the MEMS atomic vapor cell was maintained using an integrated heater. A photograph of the assembled chip-scale atomic clock prototype is shown in Figure 5b, highlighting the core optical subassembly and surrounding control electronics.

The physics package integrating the metasurface-based LP&QWP was tested using a 3596F signal analyzer and computer-controlled acquisition system. As shown in Figure 5c, the CPT microwave demodulation signal obtained with the metasurface (blue) closely matches that of the conventional LP&QWP setup (red), both exhibiting symmetric dispersive line shapes characteristic of CPT resonance. The metasurface output amplitude was within 5% of the conventional configuration, a difference within experimental uncertainty, confirming that metasurface integration does not degrade CPT performance. The slight amplitude reduction (~3–5%) arises mainly from absorption and scattering in the Cr/Ag nanostructure, while resonance contrast, slope, and noise levels remain comparable. These results demonstrate that the metasurface-based LP&QWP setup achieves equivalent CPT resonance and polarization control within a compact architecture, providing a practical replacement for bulk optics in chip-scale atomic clock systems.

**Figure 6:**
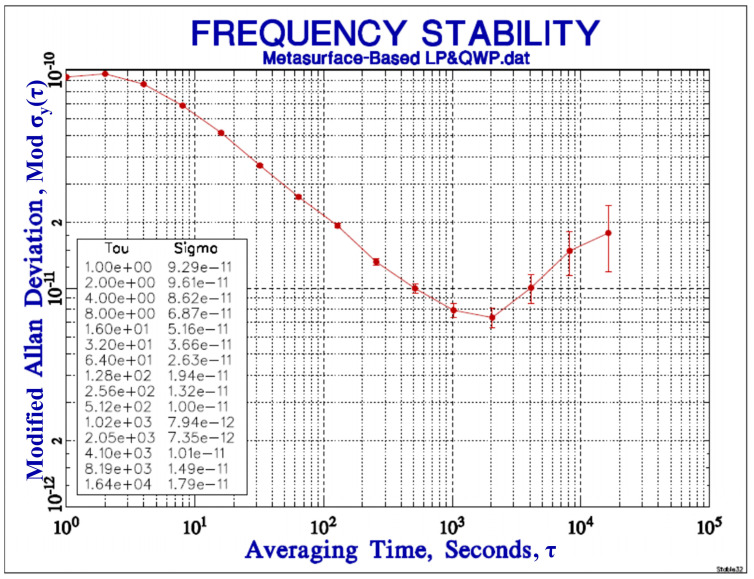
Frequency stability of the CSAC with the integrated metasurface LP&QWP.

Frequency stability was evaluated using data acquired from the 3596F analyzer and processed with the Stable32 software Version 1.62. The CSAC prototype achieved a short-term frequency stability of 9.29 × 10^−11^ at 1 s averaging time, improving to 1.59 × 10^−11^ at 10,000 s (Figure 6).

We have compared the frequency stability of the proposed chip atomic clock with other works as shown in Table 2.

These stability metrics demonstrate that the short-term stability (1 s) of our metasurface-integrated CSAC is competitive with prior works, supporting the feasibility of this approach. The long-term stability (10,000 s), though slightly lower than the best-reported value, remains within an acceptable range. Further improvements in metasurface design and system integration are planned to optimize long-term performance in future iterations.

## 5. Conclusions

In this work, we proposed and demonstrated a metasurface-based Linear Polarizer and Quarter-wave Plate (LP&QWP) for generating right-handed circularly polarized (RCP) light in highly compact Chip-scale Atomic Clocks. The polarization characteristics of the metasurface fabricated on an optical glass substrate were experimentally verified, maintaining a polarization extinction ratio (PER) of 4.8 dB and a degree of polarization (DOP) of 74.2%, confirming its ability to effectively control the state of polarization. System-level evaluation further validated the applicability of the device in a CSAC setup, underscoring the potential of nanophotonic metasurfaces and advanced nanofabrication in enabling miniaturized time-keeping systems. The measured short-term frequency stability of the CSAC incorporating the metasurface LP&QWP reached 9.29 × 10^−11^ at 1 s and 1.59 × 10^−11^ at 10,000 s averaging time. These results affirm the effective integration of the metasurface-based polarization control element into a high-stability chip-scale atomic clock platform.

## Figures and Tables

**Table 1:** Polarization results for conventional and metasurface LP&QWP modules.

Test No.	PER (dB)	DOP (%)	Polarization Type	Stability	Experimental Significance
Conventional LP + QWP	4.9	80.2	Circular polarization (high purity)	High	Closest to ideal CPT polarization state
Metasurface LP&QWP	4.8	74.2	Right-handed circular polarization	High	Closest to ideal CPT polarization state

**Table 2:** Frequency stability comparison.

	Year	Clock Type/Scheme	Frequency Stability@ 1 s	Frequency Stability@ 10,000 s	Note
Ref. [8]	2019	Glass CPT Rb Clock	6.71 × 10^−11^	2.2 × 10^−12^	Typical vapor-cell CSAC
Ref. [15]	2021	MEMS CPT Rb Clock	9.5 × 10^−11^	-	Long-term data not reported
Ref. [1]	2023	ASG CPT Rb Clock	-	1.4 × 10^−12^	Focus on long-term data
SA.45	2011	MEMS CPT Cs Clock	6.2 × 10^−11^	6.7 × 10^−12^	Typical vapor-cell CSAC Representative commercial products
XHTF 1045	2020	Glass CPT Cs Clock	8.2 × 10^−11^	1.1 × 10^−11^	Experimentally tested (this work)
This Work		Metasurface-based CPT CSAC	9.29 × 10^−11^	1.59 × 10^−11^	Comparable short-term stability, integrated LP&QWP design

## Data Availability

The data supporting the results presented in this paper are not currently available to the public but can be accessed by the authors upon a reasonable request.

## Abbreviations

The following abbreviations are used in this manuscript:

LP&QWP	Linear Polarizer and Quarter-wave Plate
CSAC	Chip-scale Atomic Clock
CPT	Coherent Population Trapping
M-PNT	Miniaturized Positioning, Navigation, and Timing
SOP	State of Polarization
MEMS	Micro Electro Mechanical Systems
VCSEL	Vertical-cavity Surface-emitting Laser
PD	Photodetector
AVCs	Atomic Vapor Cells
FDTD	Finite-difference Time-domain
RCP	Right-handed Circularly Polarized
